# Endoscopic full-thickness resection of an esophageal gastrointestinal stromal tumor without creation of a submucosal tunnel

**DOI:** 10.1055/a-2665-7193

**Published:** 2025-08-20

**Authors:** Haruhiro Inoue, Kazuki Yamamoto, Yohei Nishikawa, Ippei Tanaka, Kei Ushikubo, Mayo Tanabe, Noboru Yokoyama

**Affiliations:** 1 378609 Digestive Diseases Center, Showa Medical University Koto Toyosu Hospital, Tokyo, Japan

Gastrointestinal stromal tumors (GISTs) are rare in the esophagus, accounting for less than 2% of all cases 1 . While treatment approaches remain under discussion, submucosal tunneling endoscopic resection has shown favorable outcomes 2 . However, in the narrow submucosal space, locating the tumor without exposing its surface can be difficult, increasing the risk of capsule injury and tumor seeding. Here, we report a successful case of GIST resection using direct muscle layer incision without creating a submucosal tunnel.

A 56-year-old woman with no medical history was referred for treatment of a <3-cm mid-esophageal GIST ( Fig. 1 ). Endoscopic full-thickness resection ( Video 1 ) was planned using a GIF-H290T endoscope (Olympus) with a distal attachment (Space Adjuster; TOP) 3 , a Triangle Tip Knife J (TTJ; Olympus), and an electrosurgical unit (VIO3; ERBE, Endocut I mode: 1–3-3). After marking and submucosal injection, a mucosal incision was made to expose the muscle layer. Direct muscle dissection was then performed without tunneling ( Fig. 2 **a–d** ), enabling full-thickness resection along the tumor margin. Connective tissue between the esophageal adventitia and pericardium was meticulously detached. The tumor was then inverted into the lumen and removed en bloc ( Fig. 3 **a–d** ). The large defect, with the pericardium clearly visible, was initially approximated using the Loop-9 technique 4 , followed by complete closure with anchor-pronged clips (MANTIS; Boston Scientific). Resection was completed without capsule injury ( Fig. 4 **a–d** ).

EFTR without tunneling for esophageal GIST.Video 1

**Fig. 1:**
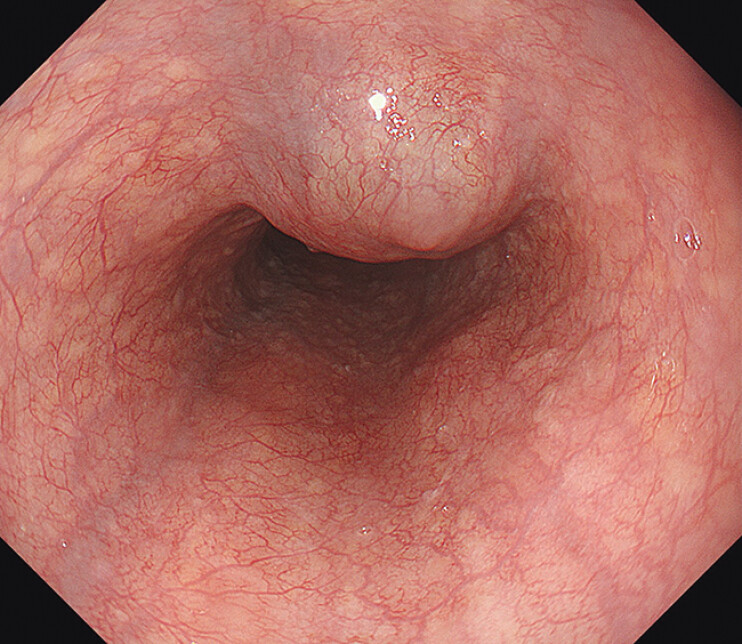
Upper endoscopy showing a submucosal tumor consistent with esophageal gastrointestinal stromal tumor located in the mid-esophagus.

**Fig. 2:**
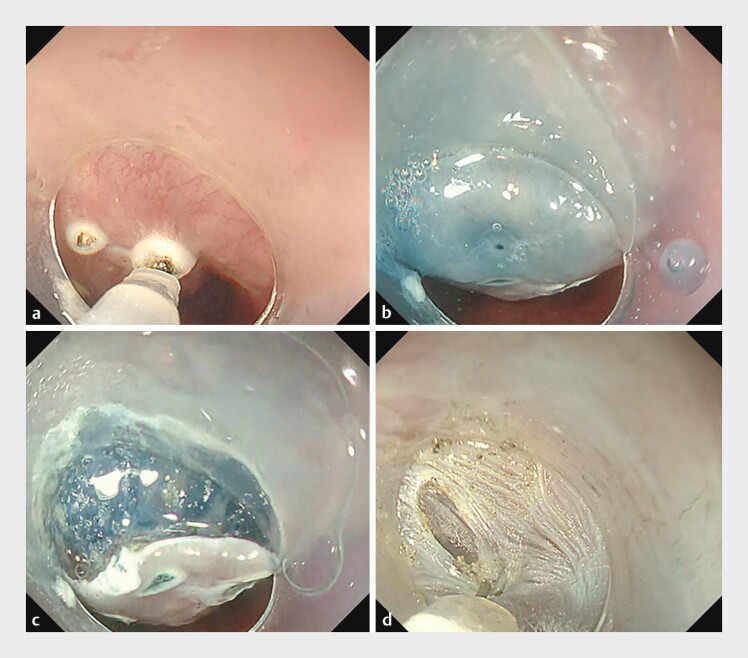
**a** Marking is performed at the proximal end of the tumor using a Triangle Tip Knife J (TTJ; Olympus). **b** Submucosal injection of saline mixed with indigo carmine is performed using a 25-G, 4-mm tip needle (NeedleMaster; Olympus). **c** A mucosal incision is made and extended to expose the underlying muscle layer. **d** Direct dissection of the muscle layer is carried out without creating a submucosal tunnel.

**Fig. 3:**
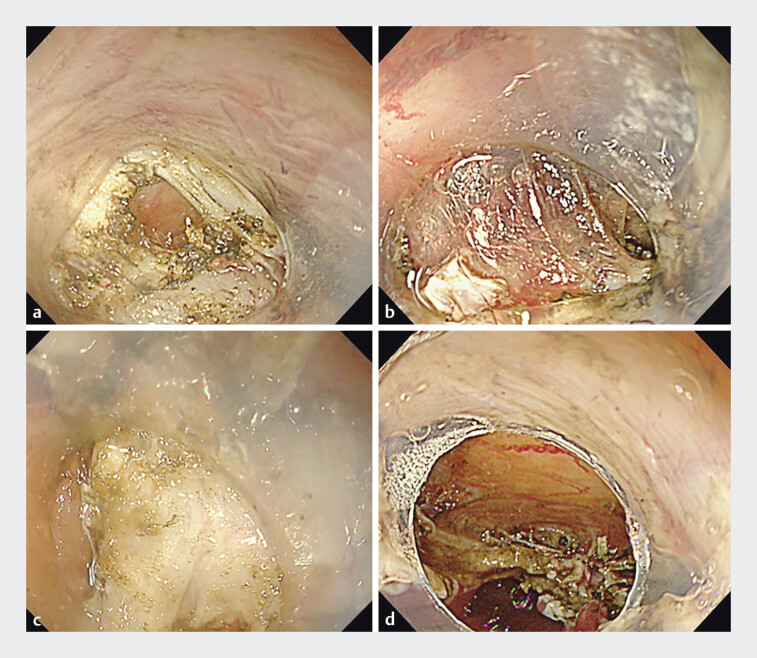
**a** Longitudinal muscle fibers are identified, and full-thickness resection is performed along the tumor margin. **b** The connective tissue between the esophageal adventitia and the pericardium is identified and carefully dissected. **c** The tumor is inverted into the esophageal lumen. **d** Full-thickness resection is completed without creating a submucosal tunnel, leaving a large defect with the pericardium clearly visible.

**Fig. 4:**
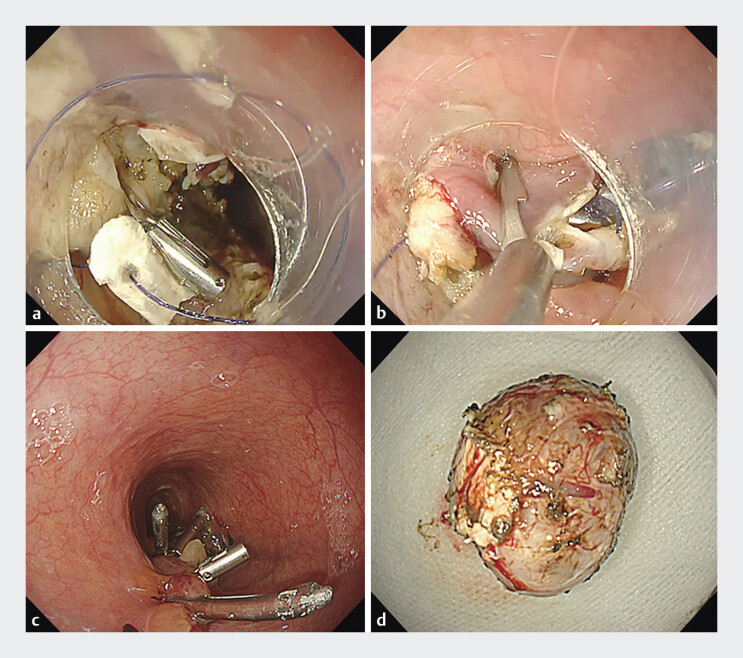
**a** Initial approximation of the large esophageal defect using the Loop-9 technique. The loop is anchored to the defect edges with clips. The distal knot is grasped using biopsy forceps inserted through the outer sheath. By simultaneously pulling the forceps and advancing the sheath, the loop is tightened. During this maneuver, the pledget functions as an anchor to facilitate closure. **b** Following the Loop-9 technique, defect closure is primarily achieved using anchor-pronged clips (MANTIS; Boston Scientific). **c** A total of six anchor-pronged clips (MANTIS; Boston Scientific) and three hemoclips (SureClip; Micro-Tech) are used to achieve complete closure of the defect. **d** En bloc resection of the gastrointestinal stromal tumor is completed without capsule injury.

The postoperative course was uneventful. Follow-up endoscopy on postoperative day 1 revealed no stricture or wound dehiscence ( Fig. 5 ). The patient resumed a regular diet and was discharged on postoperative day four as planned.

**Fig. 5:**
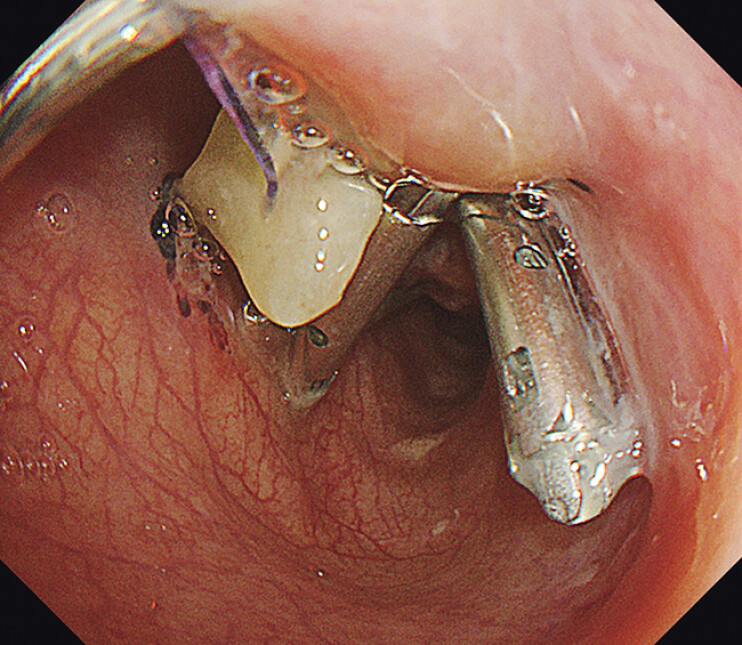
Follow-up endoscopy on postoperative day one showing no evidence of stricture or wound dehiscence.

Compared to leiomyomas, GISTs are more fragile and prone to fragmentation. Therefore, direct muscle layer incision without creating a submucosal tunnel may offer an effective approach to facilitate en bloc resection while minimizing the risk of tumor fragmentation.

Endoscopy_UCTN_Code_TTT_1AO_2AG_3AF

## Notes

Endoscopy E-Videos https://eref.thieme.de/e-videos
*E-Videos* is an open access online section of the journal *Endoscopy* , reporting on interesting cases and new techniques in gastroenterological endoscopy. All papers include a high-quality video and are published with a Creative Commons CC-BY license. Endoscopy E-Videos qualify for HINARI discounts and waivers and eligibility is automatically checked during the submission process. We grant 100% waivers to articles whose corresponding authors are based in Group A countries and 50% waivers to those who are based in Group B countries as classified by Research4Life (see: https://www.research4life.org/access/eligibility/ ). This section has its own submission website at https://mc.manuscriptcentral.com/e-videos .
